# Food-borne *Lactiplantibacillus plantarum* protect normal intestinal cells against inflammation by modulating reactive oxygen species and IL-23/IL-17 axis

**DOI:** 10.1038/s41598-020-73201-1

**Published:** 2020-10-01

**Authors:** Roberta Prete, Natalia Garcia-Gonzalez, Carla D. Di Mattia, Aldo Corsetti, Natalia Battista

**Affiliations:** Faculty of Bioscience and Technology for Food, Agriculture and Environment, University of Teramo, Teramo, Italy

**Keywords:** Cell biology, Microbiology

## Abstract

Food-associated *Lactiplantibacillus plantarum* (*Lpb. plantarum*) strains, previously classified as *Lactobacillus plantarum*, are a promising strategy to face intestinal inflammatory diseases. Our study was aimed at clarifying the protective role of food-borne *Lpb. plantarum* against inflammatory damage by testing the scavenging microbial ability both in selected strains and in co-incubation with normal mucosa intestinal cells (NCM460). Here, we show that *Lpb. plantarum* endure high levels of induced oxidative stress through partially neutralizing reactive oxygen species (ROS), whereas they elicit their production when co-cultured with NCM460. Moreover, pre-treatment with food-borne *Lpb. plantarum* significantly reduce pro-inflammatory cytokines IL-17F and IL-23 levels in inflamed NCM460 cells. Our results suggest that food-vehicled *Lpb. plantarum* strains might reduce inflammatory response in intestinal cells by directly modulating local ROS production and by triggering the IL-23/IL-17 axis with future perspectives on health benefits in the gut derived by the consumption of functional foods enriched with selected strains.

## Introduction

Over the past decades, with the rapid economic development and improvements in quality of life, our lifestyle and dietary habits have significantly changed, leading to an increasing occurrence of chronic gut inflammation and/or anomalous immune response. The human gut hosts a complex ecosystem generated by the integrity and stable cooperation between immune cells, resident microbiota and the gastrointestinal (GI) epithelium^1^, which is in charge of both organ specific and immune functions and  represents one of the major sites for generation of pro-oxidants, due to the presence of food components, microbes and direct interaction with the immune system^2^.

Oxidative stress, caused by an overproduction and accumulation of reactive oxygen species (ROS), can upregulate the expression of genes involved in adaptive and innate immune responses in the GI tract, leading to the alterations of intestinal morphology and contributing to enhance gut inflammation^3^. Currently, intervention with natural antioxidants, mainly from food sources^4^, nutrients^5^ and other bioactive components including probiotics^6,7^, has received much attention from scientists as dietary strategies to counteract oxidative stress, inflammation and some related chronic disorders^2,8^. Similarly to other natural antioxidants (i.e. plant extracts), the antioxidant role of lactic acid bacteria (LAB) has been associated with up- and down- regulation of antioxidant host functions as well as modulation of host signalling pathways^9^.

Among LAB, *Lactobacillus plantarum* (recently reclassified as *Lactiplantibacillus* (*Lpb.*) *plantarum*^10^) is a flexible and versatile species that can be found as a dominant microbiota not only in several foods, but also in the human GI tract as a natural inhabitant^11^. Beside human-derived probiotics, food-associated microbes, especially related to fermented foods, have recently recovered scientific interest for their potential health-promoting effects^12^. Previous in vitro studies have shown that *Lactobacillus* strains can modulate both oxidative stress and pro- and anti-inflammatory cytokines release^13,14^. In this context, we should recall that a redox protective effect has been recently ascribed to *Lacticaseibacillus casei* Shirota on the cellular damages induced by an oxidative stressor in an in vitro model of enterocytes^14^.

Moreover, it has been found that probiotic bacteria can reduce or even block inflammatory signalling via ROS modulation^13,15^, and some in vivo studies indicate that ingestion of probiotic LAB strains significantly modulates the oxidative stress and related inflammatory damage^7,16,17^, even though the mechanisms behind these beneficial effects are not entirely understood.

However, due to the transient condition in the GI tract, the strength of probiotics as well as of food-ingested microbes resides in sharing genes and specific metabolites, and directly interacting with epithelial and immune cells rather than in affecting persistently the microbiota composition^18^. Indeed, traditional probiotics, such as *Lactobacillus* or *Bifidobacterium,* and next-generation beneficial microbes, including *Akkermansia muciniphila*, have been reported to influence the gut barrier function^19^ and to control the secretion of different gut peptides involved in the regulation of energy metabolism^20^ via the production of short-chain fatty acids as well as to modulate gene transcription^21^.

Experimental and human studies showed that the Interleukin (IL)-23 and the downstream cytokines IL-17A and IL-17F could play a crucial role in the pathogenesis of chronic intestinal inflammation processes^22^. Experimental colitis mice models revealed IL-23 as a key cytokine that drives the intestinal inflammation^23^, whereas human studies carried out in Inflammatory Bowel Disease (IBD) patients have identified single nucleotide polymorphisms in many genes encoding for proteins involved in the IL-23/IL-17 pathway^24^ as well as increased levels of IL-23 and IL-17 cytokines^23^. Based on that, IL-23/IL-17 inflammation pathway has been proposed as a novel therapeutic target in IBD and other gastrointestinal disorders^25^ and interestingly, IL-17/IL-23 axis inhibition by *Lactobacillus* commensal bacteria showed an amelioration of DSS-induced colitis symptoms^26,27^, suggesting putative alternative treatment strategy.

**Figure 1:**
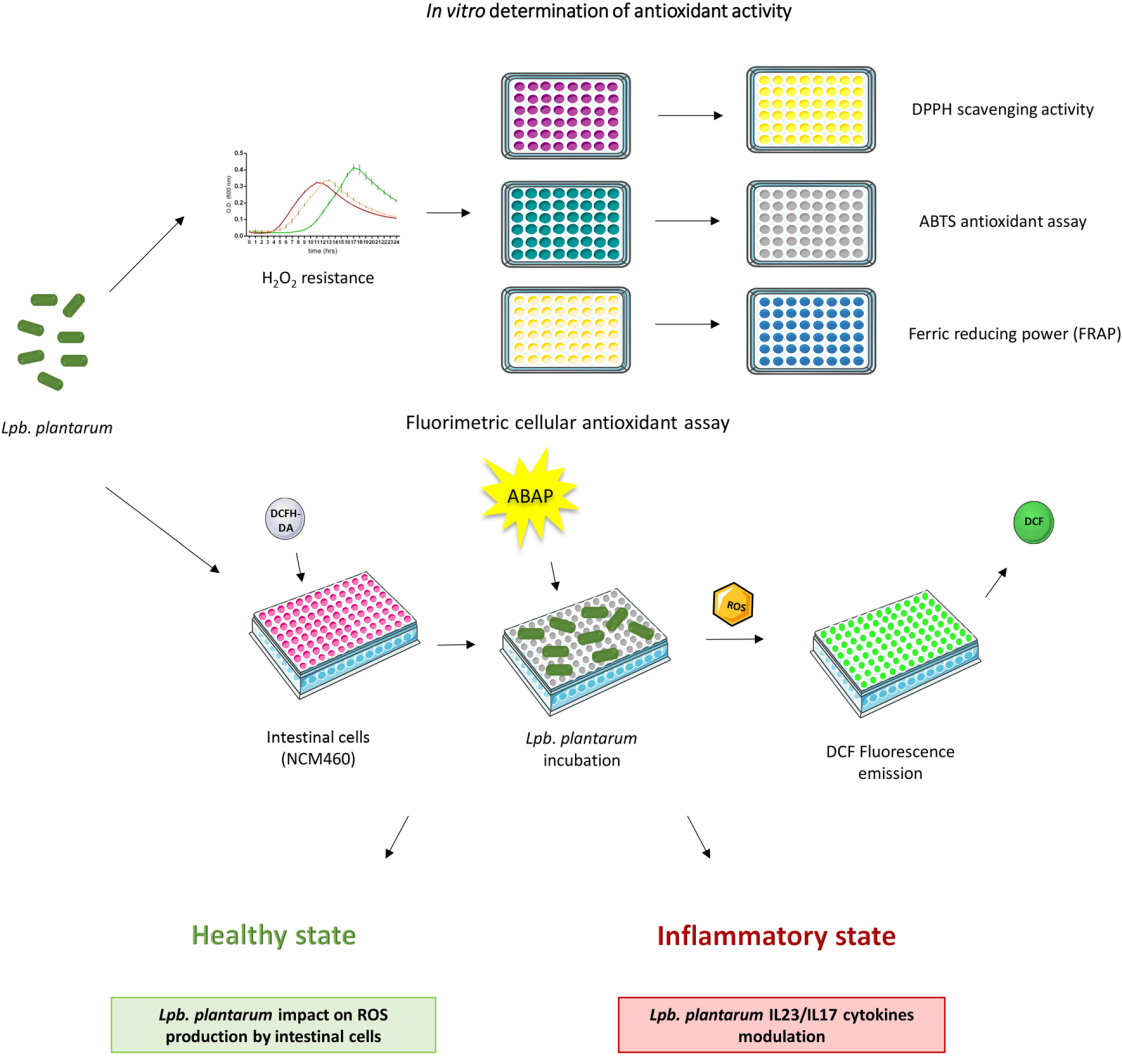
Graphical scheme showing the workflow for in vitro determination of *Lpb. plantarum* antioxidant activity and *Lpb. plantarum* differential impact on intestinal cells, reported in the study. Graphical illustrations were created by using some graphical elements from Servier Medical Art by Servier, available on https://smart.servier.com/ under a Creative Commons Attribution 3.0 Unported License.

These findings prompted us to elucidate the protective role of previously selected food-associated *Lpb. plantarum* strains, already characterized for several properties^28–31^, to face oxidative stress and related inflammatory damage at intestinal level. For this purpose, food-associated and human *Lpb. plantarum* strains (Table 1) were examined for their in vitro capacity to tolerate oxidative stress as well as for their antioxidant potential by three different microplate chemical assays (DPPH (1,1-diphenyl-2-picrylhydrazyl),  (2,2-azino-bis(3-ethylbenzothiazoline-6-sulfonic acid) and FRAP (ferric reducing antioxidant power)). In addition, the specific ability of each strain to modulate ROS levels in response to either oxidative or inflammatory stress and to reduce IL-17A, IL-17F and IL-23 release in an inflamed intestinal cell model was examined. Figure 1 shows the workflow for in vitro determination of *Lpb. plantarum* antioxidant activity and *Lpb. plantarum* differential impact on intestinal cells reported in the study.

**Table 1:** *Lpb. plantarum* strains used in the study.

Strain	Origin	Source
WCFS1	Human saliva	Reference strain, UNITE collection
IMC513	Human gut	Probiotic strain, Synbiotec srl
O13, C9O4	Table olives	UNITE collection
LT52, LT100	Raw-milk cheeses	UNITE collection

## Results

### Antioxidant activity of *Lpb. plantarum* strains

**Figure 2:**
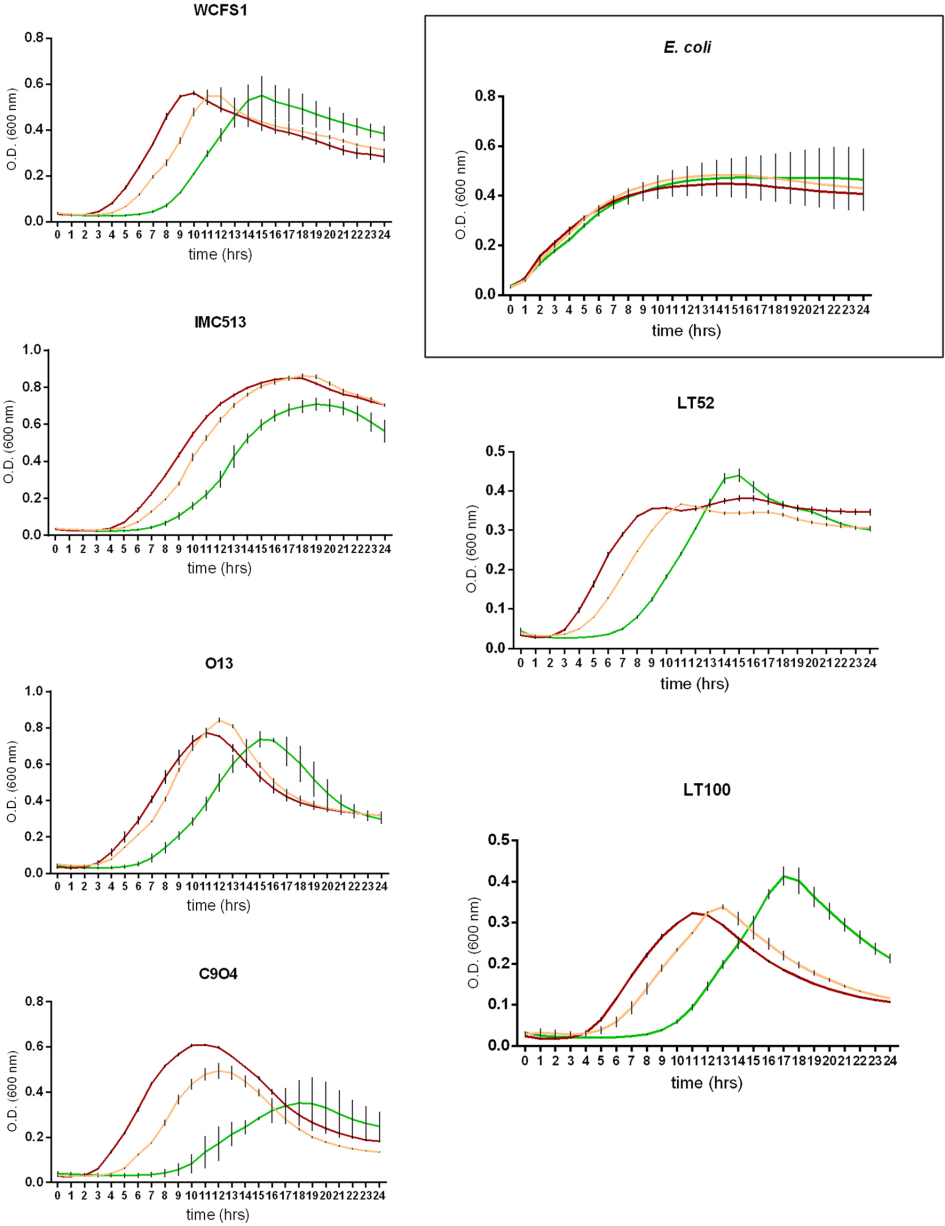
*Lactiplantibacillus plantarum* survival in presence of hydrogen peroxide (red line means MRS broth, yellow line means MRS broth with 5.0 mM hydrogen peroxide, green line means MRS with 10 mM hydrogen peroxide). *Escherichia coli* was used as catalase positive reference strain.

The ability of food-associated and human-derived *Lpb. plantarum* strains to tolerate oxidative stress was assessed in the presence of hydrogen peroxide by monitoring microbial growth for 24 h. As shown in Fig. 2, *Lpb. plantarum* growth was not significantly affected in presence of low concentration of hydrogen peroxide (with the exception of C9O4), whereas the higher concentration highlighted a strain-dependent behaviour. In all *Lpb. plantarum* strains, 10 mM hydrogen peroxide influenced the microbial growth by causing an extension of the lag phase, whereas, among all the strains tested, a higher cell density was showed by the food-associated strains LT52 and LT100 at the end of the exponential phase. Therefore, all *Lpb. plantarum* strains were able to endure levels of induced oxidative stress much higher than the levels usually tested in the well-known semi quantitative method of Buchmeier and co-workers^32^. In this context, it should be noted that *Escherichia coli*, used as catalase positive reference strain, showed no appreciable growth inhibition in presence of both hydrogen peroxide concentrations (Fig. 2).

The potential antioxidant activity of food-borne *Lpb. plantarum* strains was tested by three different in vitro assays (ABTS, DPPH and FRAP), all of them optimized and adapted to a microplate format. These methods were chosen since, as they differ in several aspects such as the mechanism of action (ABTS, DPPH are based on radical reactions while FRAP on a redox one) and the environmental conditions (solvent polarity and pH), they can provide an insight into the in vitro antioxidant capacity of the strains under investigation. As shown in Table 2, *Lpb. plantarum* cells showed an overall higher ability to scavenge the  radical cation compared to the DPPH method, in which the inhibition percentage was around two-fold lower, for the majority of strains. In particular, *Lpb. plantarum* LT100 (isolated from raw-milk cheeses) displayed a high value of antioxidant activity (48.9 ± 1.74) in term of  inhibition percentage, similar to that of IMC513 (from human source), as reported in Table 2. Regarding the reducing power of *Lpb. plantarum*, the table olives-associated O13 strain showed the highest FRAP value (209.6 ± 4.70 mmol Fe^2+^/ml), higher than the strains WCFS1 (163.3 ± 11.08 mmol Fe^2+^/ml) and IMC513 (134.9 ± 6.43 mmol Fe^2+^/ml). Therefore, the combinations of all these results suggested the potential ability of some *Lpb. plantarum* strains to partially neutralize free radicals by different mechanisms with variations in a strain-dependent manner.

**Table 2:** Determination of antioxidant activity of *Lpb. plantarum* by ABTS, DPPH and FRAP methods.

Strains	(%)	DPPH (%)	FRAP (mmol Fe^2+^/ml)
WCFS1	59.4 ± 0.43^a^	20.1 ± 1.21^a^	163.3 ± 11.08^d^
IMC513	41.6 ± 1.93^b^	20.1 ± 0.98^a^	134.9 ± 6.43^c^
O13	30.2 ± 2.91^c^	14.4 ± 2.58^b^	209.6 ± 4.70^a^
C9O4	29.3 ± 1.36^c^	24.6 ± 0.85^a^	97.26 ± 1.80^b^
LT52	32.4 ± 1.49^c^	17.3 ± 0.59^a^	131.7 ± 3.34^c^
LT100	48.9 ± 1.74^b^	19.5 ± 3.07^a^	101.8 ± 2.09^b^

^a–c^Mean values with the same superscript are not different (*p* > 0.05) by ANOVA Bonferroni’s test.

### ROS modulation by *Lpb. plantarum*

**Figure 3:**
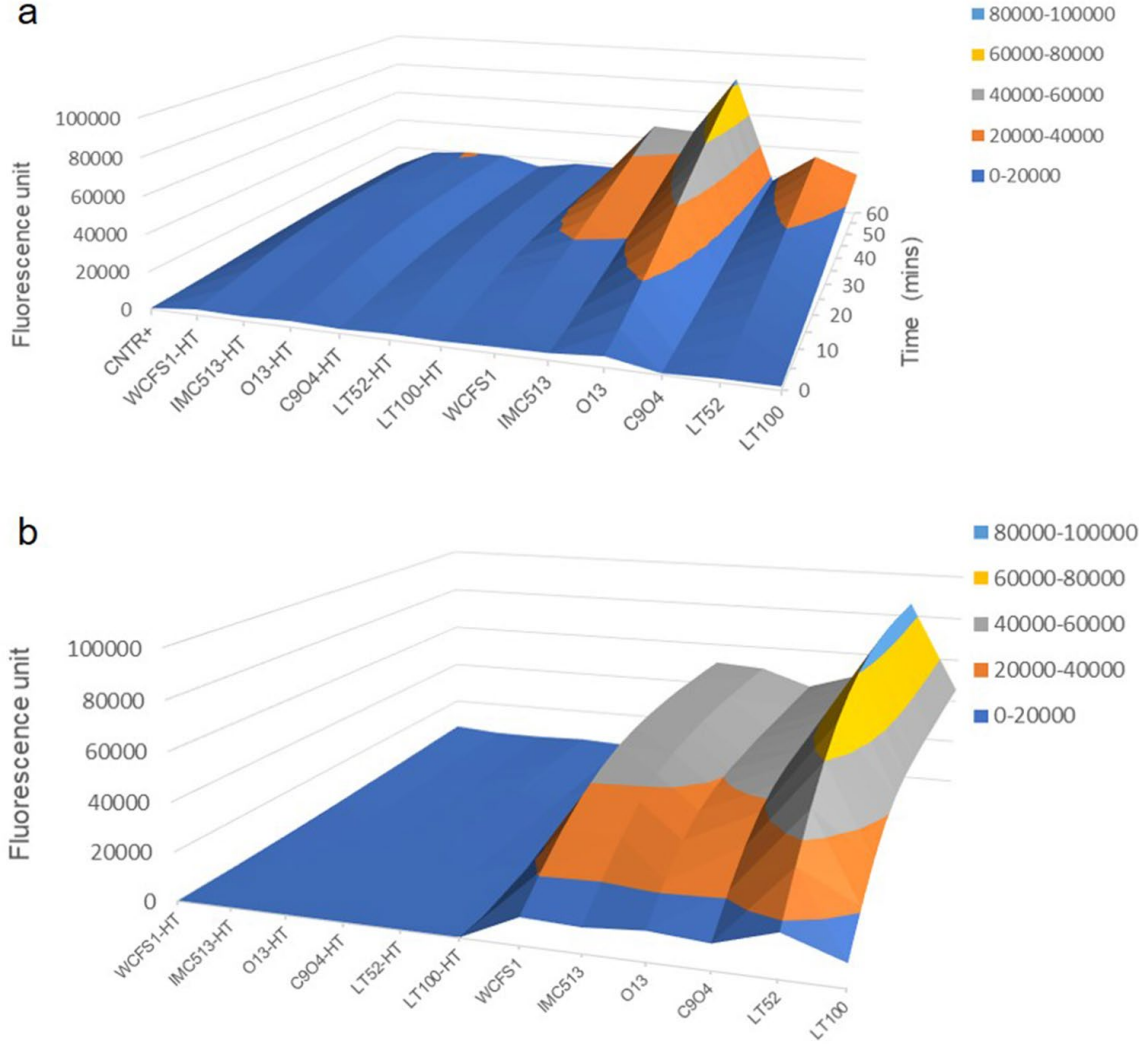
ROS modulation by *Lactiplantibacillus plantarum* strains. (**a**) Peroxyl radical-induced oxidation of DCFH to DCF in NCM460 cells by live and heat-treated (HT) *Lpb. plantarum* strains over time. (**b**) *Lpb. plantarum* live and heat-treated (HT) cells response to the fluorescence probe DCFH-DA (25 µM) over time.

**Figure 4:**
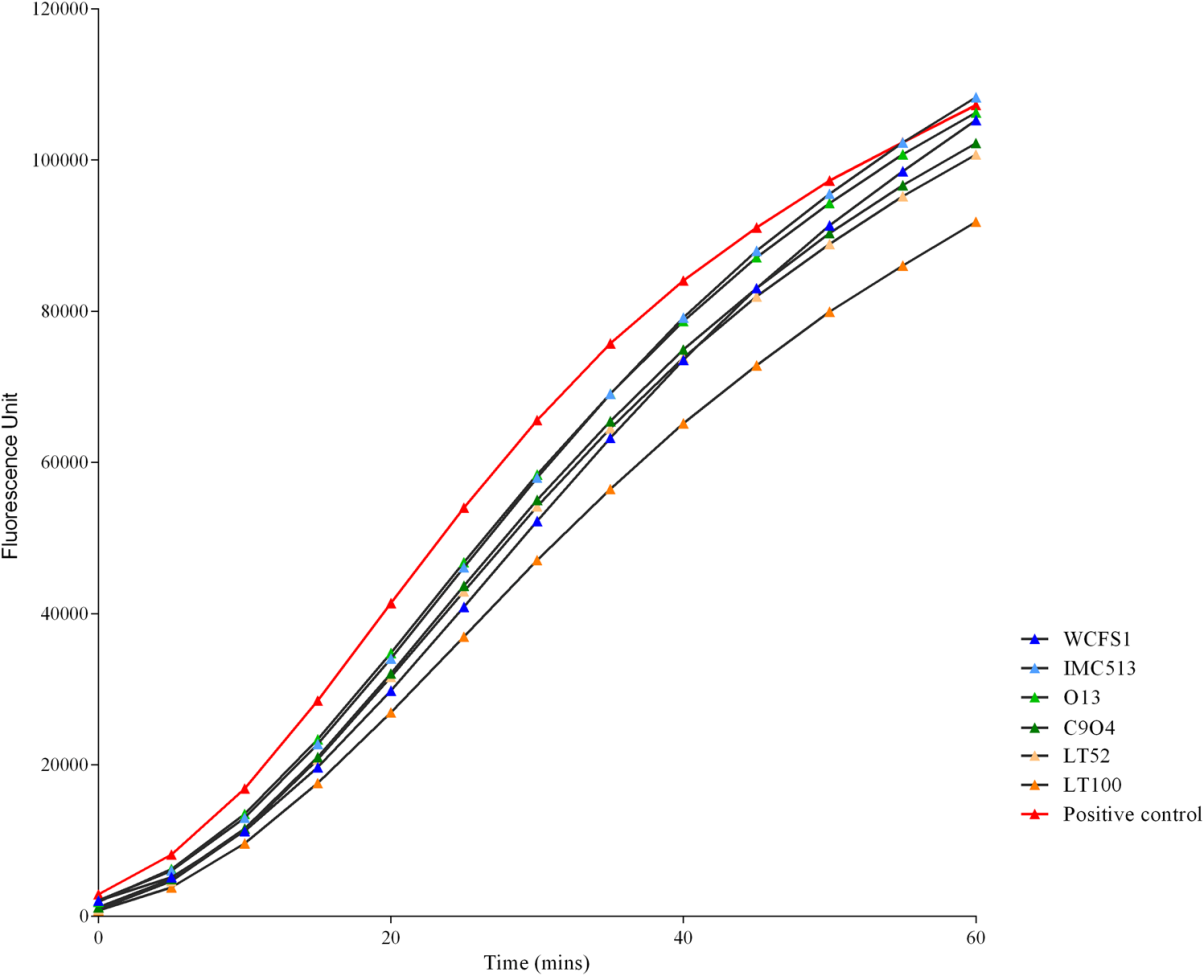
ROS production by inflamed NCM460 cells over time, after pre-treatment with live *Lpb. plantarum* strains. Data of one representative experiment are reported in the graph.

Based on the above described ability to either endure induced oxidative stress or partially neutralize ROS, we tested the potential protective impact of four food-associated *Lpb. plantarum,* besides the two human-derived strains WCFS1 and IMC513, on both normal and inflamed NCM460 cells. In order to confirm ROS production in response to the oxidative treatment with 2,2'-azobis (2-aminidopropane) dihydrochloride solution (ABAP) and to evaluate the impact of *Lpb. plantarum* strains on cellular ROS levels, the non fluorescent probe 2′,7′-dichlorofluorescein diacetate (DCFH-DA), that in presence of ROS inside the cells is oxidized into the highly fluorescent dichlorofluorescein (DCF), was used to determine ROS generation in NCM460 preliminarily co-incubated with live and heat-treated (HT) bacterial cells. Interestingly, intestinal cells pre-treated with live *Lpb. plantarum* cells showed increased ROS levels in response to induced oxidative stress over 1 h exposure to ABAP, while inactivated HT bacterial cells did not display any effect on ROS production, as clearly shown by the surface chart in Fig. 3a. In particular, *Lpb. plantarum* O13 isolated from table olives showed the highest ROS generation over time (up to 2.5-fold compared to the control). To confirm the different behaviour of live and heat-inactivated *Lpb. plantarum* cells on NCM460 cells, their response to the fluorescence DCF probe was also tested in the absence of intestinal cells (Fig. 3b). The surface chart in Fig. 3b displays a similar response compared to Fig. 3a, confirming that live cells are needed to exert a potential effect on human cells. Moreover, in order to evaluate whether *Lpb. plantarum* strains can reduce inflammatory signalling via ROS modulation, we investigated the *Lpb. plantarum* impact on ROS production in an in vitro inflammation model^29^. Compared to the positive control (inflamed intestinal cells) pre-treatment with all food-associated *Lpb. plantarum* strains resulted to be effective in the reduction of ROS levels, generated by intestinal cells after 24 h of exposure to the inflammatory stimulus. They also showed a similar ability of human strains WCFS1 and IMC513 to modulate human cells anti-inflammatory responses (Fig. 4).

### Protective impact on cytokine release in inflamed intestinal cells by *Lpb. plantarum*

**Figure 5:**
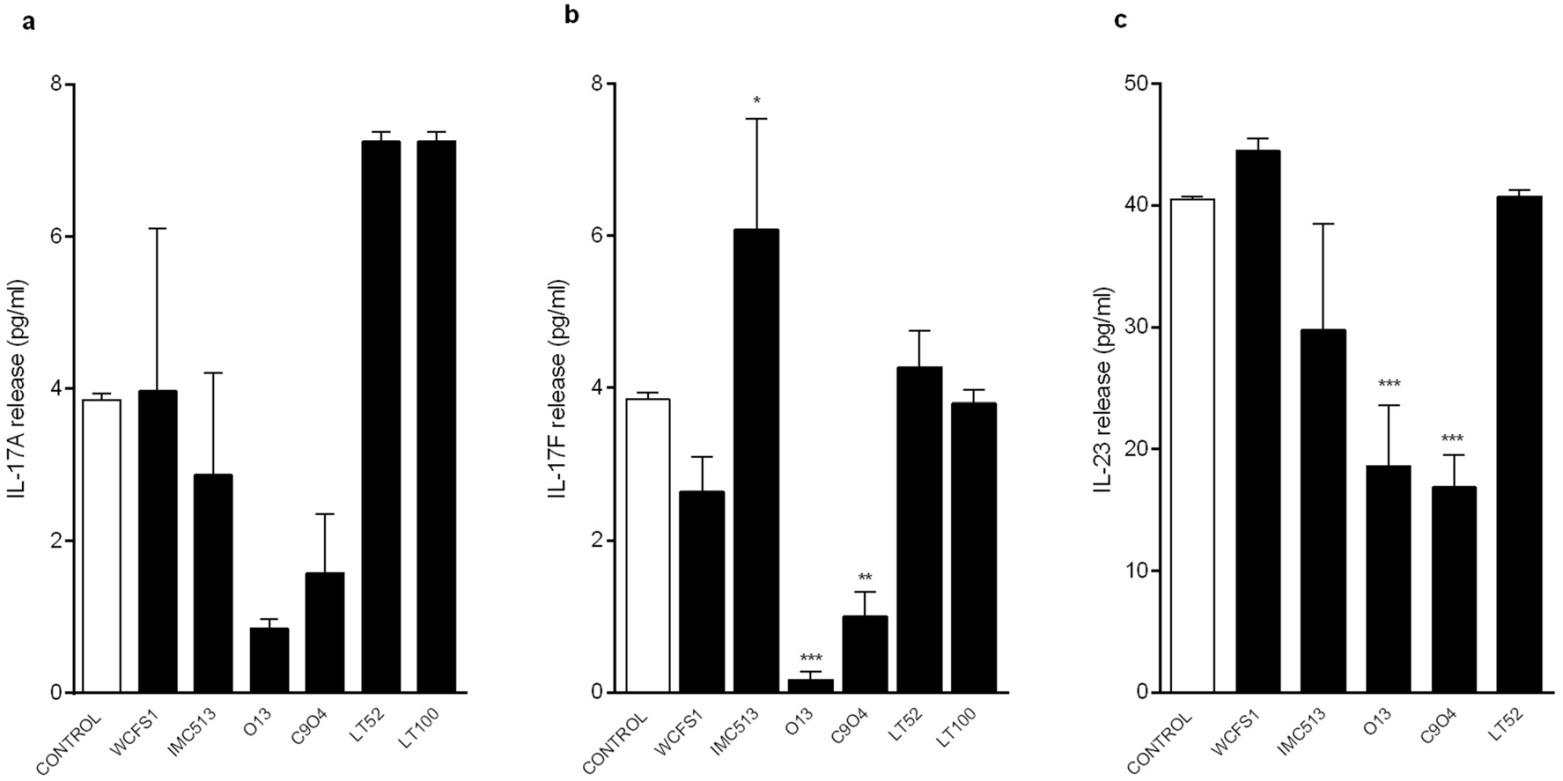
*Lpb. plantarum* modulation of (**a**) IL-17A, (**b**) IL-17F and (**c**) IL-23 release on inflamed NCM460 cells. ***p < 0.0002.

Figure 5 shows the overall ability of *Lpb. plantarum* to modulate pro-inflammatory cytokines IL-17A, IL-17F and IL-23 levels in our inflamed cell model. Among all the tested strains, *Lpb. plantarum* O13 and C9O4 significantly reduced IL-17F (0.17 pg/ml and 1.00 pg/ml, respectively) and IL-23 (18.6 pg/ml and 16.9 pg/ml, respectively) levels compared to the control (3.85 pg/ml for IL-17F and 40.5 pg/ml for IL-23), and a similar, but not significant trend, was also observed in IL-17A reduction (Fig. 5). Overall, these findings suggest that our *Lpb. plantarum* might affect in a strain-dependent manner the potential key role of IL-23/IL-17 inflammation axis in driving the intestinal inflammation.

## Discussion

Modulation of host immunity and stimulation of host defence systems through anti-inflammatory and antioxidant responses are the most claimed beneficial effects of both commensal and probiotic bacteria mutualistic interactions with the human host^33^. In the last years, *Lpb. plantarum* strains have been studied not only for their functional traits, but also for their demonstrated health-promoting properties^11,34^. However, there is a lack of investigation on evaluating the impact of food-ingested *Lpb. plantarum* strains, which are likely to be consumed at high concentrations in fermented foods such as table olives ^35^. Indeed, in fermented foods they are one of the most predominant species, depending on their innate capability to overcome spontaneous developing microbiota or as a consequence of a deliberately addition to confer new functionality to fermented foods^34^,^36^. In view of their promising potential properties, here we investigated four selected food-borne and two human derived *Lpb. plantarum *strains, to test both their in vitro antioxidant activities and their ability to reduce the inflammatory response via ROS modulation in a recently reported in vitro cell model that mimics inflammatory conditions^29^.

The molecular mechanisms of *Lpb. plantarum* antioxidative response is still not entirely understood, and it has been previously shown that some lactobacilli counteract induced oxidative stress in different manners^37,38^. For this reason, we applied a combined approach of in vitro techniques to determine antioxidant activity in terms of direct free radicals neutralization via hydrogen or electron-transfer, ferric reducing power and resistance to hydrogen peroxide (H_2_O_2_). Firstly, growth curves in the presence of 5 mM and 10 mM H_2_O_2_ were performed in order to assess the ability of *Lpb. plantarum* strains to endure induced oxidative stress. It emerged that all of the *Lpb. plantarum* strains tested remained viable in the presence of 10 mM H_2_O_2_ with a clear strain-dependent behaviour. This is in contrast to the study of Tang and co-workers who showed that a concentration of 2.5 mM of H_2_O_2_ completely inhibited the growth of the *Lpb*. *plantarum* strain MA2^39^. However, as it has been previously reported^39^, the oxidative environment does markedly influence the growth of the strains, causing an extension of the lag phase without any killing effect, likely as a result of the initial stress conditions. Moreover, during the exponential phase two food-associated strains (LT52 and LT100) showed a strong recovery of the growth rate, with optical density (OD) values much higher than those of the control (without H_2_O_2_), revealing a potential inducible repair system. Even though *Lpb. plantarum* does not have a complex regulation system to defend against oxidation as eukaryotic cells, the presence of some enzymes (such as NADH-dependent enzymes and superoxide dismutase), the production of antioxidant metabolites (folate and glutathione) and exopolysaccharides are regarded as important defence mechanisms to face oxidative stress among lactobacilli^9,40,41^.

Overall results from the in vitro assays DPPH, ABTS and FRAP, showed that *Lpb. plantarum* strains displayed strong and strain-dependent antioxidant activity, mainly characterized by their relevant ferric reducing power (Table 2). This potent ability of chelating metal ions such as Fe^2+^ has been previously described for other lactobacilli strains^42^. Moreover, results of direct free radicals neutralization showed that food-associated *Lpb. plantarum* strains displayed strong antioxidant activity, with an overall higher ability to scavenge the  radical cation compared to the DPPH method, in which the inhibition percentage was around two-fold lower for the majority of strains (Table 2). Regardless of this, the observed DPPH free radical scavenging activity of *Lpb. plantarum* strains in this study are comparable to the levels obtained by Li and co-workers^42^. Indeed, they found the DPPH free radical scavenging activity of selected *Lpb. plantarum* strains, when measured at 10^9^ CFU/ml, are comparable to levels obtained for other lactobacilli^38^ and to our results, with values ranging from 15–20% (Table 2). In general, for those strains with higher antioxidant activity, we can ascribe a correlation among the different in vitro test performed. This is the case of the food-associated *Lpb. plantarum* LT100 strain, that shows the highest value of antioxidant activity in term of  inhibition percentage, and is also one of the most resistant strains to hydrogen peroxide-induced oxidative stress, as well as the strains WCFS1 and IMC513.

Although these chemical assays are widely applied methods for testing antioxidant activity, they may not reflect the actual biological activity of bacteria inside human cells^43^. Therefore, we tested the antioxidant activity of *Lpb. plantarum* strains through the cellular DCFH-DA assay, a more biologically representative method, largely applied to assess microbial ROS modulation in different cell lines^14,43–45^. Interestingly, the results showed that ROS modulation by *Lpb*. *plantarum* strains is markedly influenced by the health status of the intestinal cells. Whilst a potential preventive role of *Lpb. plantarum* was observed with a healthy intestinal cell model by increasing ROS production (Fig. 3), the data obtained with the inflamed intestinal cells indicate a potential protective role in ameliorating inflammation conditions by decreasing ROS release (Fig. 4). Our results are in agreement with several reports showing that the administration of probiotics promotes the development of some cellular antioxidant defence mechanisms in different pathological and inflamed enterocytes-like cell models^37,43,44^. This relationship between inflammatory status of the cells and oxidative stress has been previously documented by other investigators^46–49^. In view of the pivotal role of cytokines in modulating oxidative stress and the potential of probiotic bacteria to reduce or even block inflammatory signalling via ROS modulation, we investigated the ability of our strains to trigger the IL-17/IL-23 axis in the inflamed intestinal cell model. In accordance with other studies, in which it has been demonstrated that the down-regulation of the IL23/Th17 pathway could ameliorate chronic inflammatory symptoms^14,15^, we observed that two strains isolated from table olives, O13 and C9O4, in addition to reducing ROS production in inflamed cells, significantly decreased IL-17F and IL-23 levels compared to the control, whereas a similar, but not significant trend, was also observed in IL-17A reduction (Fig. 5).

**Figure 6:**
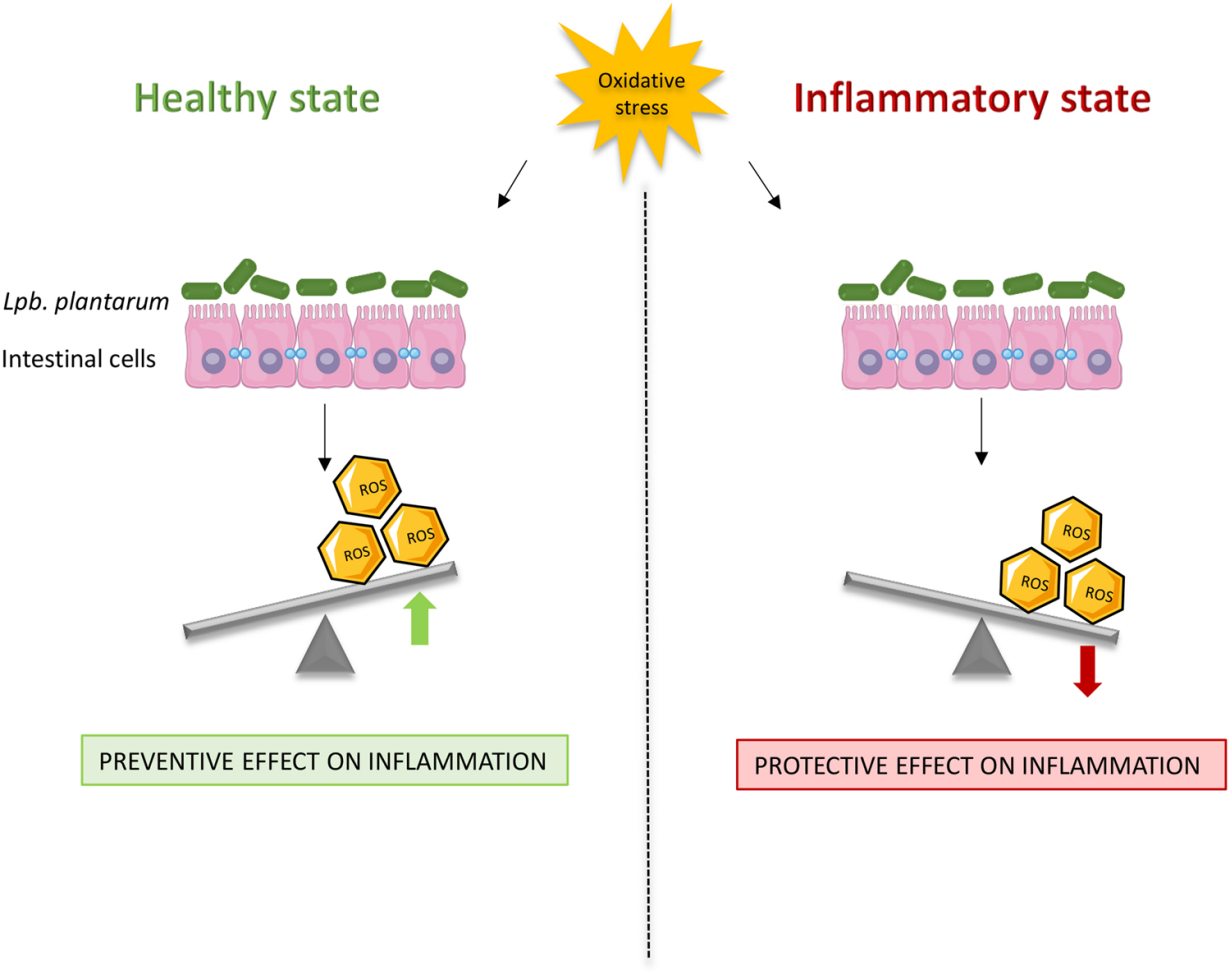
Schematic representation of the speculated effects of *Lpb. plantarum* on ROS production by healthy and inflamed intestinal cells upon oxidative stress. Graphical illustrations were created by using some graphical elements from Servier Medical Art by Servier, available on https://smart.servier.com/ under a Creative Commons Attribution 3.0 Unported License.

The interaction of microorganisms with the host, together with their anti-oxidative and anti-inflammatory potential role, can occur through different mechanisms of action, depending on a wide range of factors, such as physiological and/or pathological conditions as well as individual strain activity. Although the precise determination of the complex microbe-host relationship is still a hard-scientific challenge, this in vitro study suggests a differential impact of *Lpb. plantarum* on ROS production by healthy and inflamed intestinal cells upon oxidative stress (Fig. 6), opening a promising scenario for further investigations.

In conclusion, this study evidences that our *Lpb. plantarum* strains are able to endure levels of induced oxidative stress through the modulation of ROS and IL-23/IL-17 axis, suggesting a promising environmental fitness for their potential use as a personalized probiotic supplement tailored for the benefit of patients affected by GI disorders. Further in vivo experimental animal studies are needed to clarify and validate the beneficial contribution of our *Lpb. plantarum* strains to overcome the limits due to an in vitro approach. However, we must recall in this context the warning of the United States Environmental Protection Agency to stop the studies on mammals by 2035, reinforcing thus the use of innovative in vitro models to translate the health benefits observed during research into real-life outcomes^50^. Additionally, since these strains can be commonly ingested with foods, such as table olives with a recognized antioxidant capability due to high polyphenols content^35^ they could beneficially affect the consumer by providing another dietary source of natural antioxidants or by exerting a potential protective role in the GI tract to counteract gut inflammation and oxidative disorders.

## Methods

### Bacterial strains

*Lpb. plantarum* strains investigated in this study were selected among our laboratory collection at the University of Teramo (Table 1). All *Lpb. plantarum* strains were previously isolated from different sources as well as characterized for several properties including their potential ability to survive and interact in an in vitro cellular model^28–31^. *Lpb. plantarum* WCFS1 and a commercial probiotic strain, *Lpb. plantarum* IMC513 (kindly provided by Synbiotec s.r.l., Camerino, MC, Italy), were included in the study as human-derived reference strains (Table 1). All the strains were routinely grown under microaerophilic conditions using de Man, Rogosa and Sharp (MRS) medium (Oxoid Ltd, Basingstoke, United Kingdom) at 37 °C. *Lpb. plantarum* strains were grown in MRS broth at 37 °C for 8 h. Subsequently, bacterial cells in exponential growth phase were harvested by centrifugation (14,000 rpm, 10 min, 4 °C), washed twice with sterile phosphate buffer saline (PBS) and resuspended in sterile PBS at 10^9^ CFU/ml, before each assay. In order to assess *Lpb. plantarum* impact on ROS production by intestinal cells, experiments were carried out using both live and heat treated (HT) cells at 100 °C for 30 minutes^51^.

### Intestinal cell culture

Normal human colon mucosal epithelial (NCM460) cells (INCELL Corporation, LLC, Sant’Antonio, TX, USA) were grown in INCELL’s enriched M3Base medium supplemented with 1% (v/v) Penicillin/Streptomycin 100 × (Corning, NY, United States), 1% (v/v) Non-Essential Amino Acids 100 × solution (Corning, NY, United States), and 10% (v/v) heated inactivated Fetal Bovine Serum (FBS; Corning, NY, United States). Cells were grown in culture dishes at 37 °C in a 5% CO_2_ atmosphere, and seeded at 60–70% confluence (10^5^ cells/well in 96-well plate) for 24 h prior to co-incubation with the bacterial strains.

### In vitro determination of antioxidant activity of *Lpb. plantarum* strains (chemical assays)

#### Resistance to hydrogen peroxide

Microbial survival rate under oxidative stress was assessed for each strain by monitoring growth in presence of hydrogen peroxide (Sigma Aldrich, St Louis, USA). Briefly, *Lpb. plantarum* strains were incubated in MRS broth containing 5 mM and 10 mM of hydrogen peroxide (30% wt. solution, Sigma Aldrich, St Louis, USA) at 37 °C for 24 h. Bacterial cell growth was monitored at 600 nm using an EnSpire multimode plate reader (PerkinElmer, Waltham, MA, United States). The plate reader was run in discontinuous mode with absorbance readings performed in 60 min intervals before 30-s shaking at medium speed. Cultures were grown in three biologically independent replicates and the resulting growth data were expressed as the mean of these replicates. *Escherichia coli*, used as catalase positive reference strain, was prepared and tested as *Lpb. plantarum* strains by incubation in Nutrient broth (Oxoid Ltd, Basingstoke, United Kingdom).

#### Antioxidant capacity using ABTS method

A microplate format of the  [2,2-azino-bis(3-ethylbenzothiazoline-6-sulfonic acid)] radical cation method^52^ was optimized and used to assess the antioxidant activity of *Lpb. plantarum* strains. ABTS stock solution (7 mM) was mixed with 2.45 mM potassium persulfate to produce ABTS radical cation ( ) and the mixture was stored in the dark at room temperature for 12-16 h. Before use,  working solution was prepared by diluting  solution in PBS to adjust the absorbance at 734 nm to 0.9 ± 0.0.02. The assay was performed by adding 0.25 ml of either each strain suspension or PBS (used as control) in 1.0 ml of  working solution. After 5 min at room temperature, each sample was harvested by centrifugation (14,000 rpm, 5 min, 4 °C) to remove bacterial cells. A volume of 0.2 ml/well was used for each sample, blank (0.2 ml PBS) and control, thus absorbance readings from three independent biological replicates were carried out by using an EnSpire multimode plate reader (PerkinElmer, Waltham, MA, United States). Antioxidant activity of each strain is expressed as:

$${\text{Percentage }}\;{\text{inhibition}}\; \, \left( \% \right) \, = \, \left[ {\left( {{\text{A}}_{{\text{c}}} - {\text{A}}_{{\text{s}}} } \right)/{\text{A}}_{{\text{c}}} } \right] \, \times { 1}00$$

where A_c_ is the absorbance of the control, A_s_ is the absorbance of  after co-incubation with each *Lpb. plantarum* strain.

#### Scavenging ability on DPPH (1,1-diphenyl-2-picrylhydrazyl) free radical

The DPPH radical-scavenging capacity of *Lpb. plantarum* strains was determined by optimizing the method described by Wang and co-workers^53^ to a microplate format. Briefly, 0.5 ml of each *Lpb. plantarum* suspension was mixed with 1.0 ml of 0.2 mM DPPH ethanolic solution and was allowed to stand in the dark for 30 min at room temperature. Subsequently, each sample was harvested by centrifugation (14,000 rpm, 5 min, 4 °C) to remove bacterial cells. Equally, a same proportion (0.5 ml) of PBS was added to the DPPH solution and use as control. A volume of 0.2 ml/well was used for each sample, blank (0.2 ml absolute ethanol) and control, then absorbance readings at 517 nm from three independent biological replicates were recorded by using an EnSpire multimode plate reader (PerkinElmer, Waltham, MA, United States). Antioxidant activity of each strains is expressed as:

$${\text{Percentage }}\;{\text{inhibition }}\left( \% \right) \, = \, \left[ {\left( {{\text{A}}_{{\text{c}}} - {\text{A}}_{{\text{s}}} } \right)/{\text{A}}_{{\text{c}}} } \right] \, \times { 1}00$$

where A_c_ is the absorbance of the control, A_s_ is the absorbance of DPPH after co-incubation with each *Lpb. plantarum* strain.

#### Ferric reducing antioxidant power (FRAP)

The ferric reducing antioxidant power (FRAP) was assessed by a microplate format of FRAP assay^54^. FRAP working-solution was prepared daily by mixing 10-volumes of acetate buffer (300 mM, pH 3.6) with 1-volume of 2,4,6-Tripyridyl-s-Triazine (TPTZ, 10 mM dissolved with 40 mM HCl) and 1-volume of ferric chloride (20 mM in water). The assay was performed by adding 0.2 ml of *Lpb. plantarum* cultures (or PBS as blank) to 0.8 ml FRAP working-solution, pre-warmed at 37 °C. The mixtures were incubated in the dark at 37 °C for 30 min. After removing bacterial cells by centrifugation (14,000 rpm, 5 min, 4 °C), absorbance readings from three independent biological replicates were recorded at 593 nm by using an EnSpire multimode plate reader (PerkinElmer, Waltham, MA, United States). FeSO_4_·7H_2_O solutions, in the range 100-1000 µmol/liter, were used to graph a calibration plot, and the reducing activity of each strain was expressed as mmol/ml of Fe^2+^.

### Assessment of potential protection of *Lpb. plantarum* strains on intestinal cell model

#### *Lpb. plantarum* impact on ROS production by intestinal cells

The *Lpb. plantarum* modulation of ROS levels in both normal and inflamed intestinal cell model was investigated by a fluorimetric microplate dichlorofluorescein diacetate (DCFH-DA) assay^43^. Briefly, normal NCM460 cells were incubated with 25 μM DCF-DA dissolved in Hanks’ Balance Salt Solution (HBSS) for 1 h at 37 °C, then washed twice with HBSS and incubated with live and heat-treated (100 °C, 30 min) *Lpb. plantarum* strains for 1.5 h at 37 °C. Subsequently, 0.1 ml of 600 µM of 2,2′-azobis (2-amidinopropane) dihydrochloride solution (ABAP) was added as free radical generator. 2′,7′ dichlorofluorescein (DCF) fluorescence was monitored every 5 min for 1 h by using an EnSpire Multimode Plate Reader (PerkinElmer, Waltham, MA, USA) at excitation and emission wavelengths of 485 and 535 nm, respectively. DCFH-DA assay was also carried out to assess the *Lpb. plantarum* live and HT cells response to the fluorescence probe DCFH-DA over time. For each single experiment, HBSS fluorescence values were used as blank, whereas cells treated with DCFH-DA and ABAP were used as positive control, and results are expressed as fluorescence unit over time.

#### *Lpb. plantarum* cytokines modulation

To evaluate *Lpb. plantarum* cytokines modulation on inflamed NCM460 cells, IL-17F and IL-23 cytokine production were detected through an extremely sensitive high-throughput method for multiplex protein analysis (LUNARIS Technology, AYOXXA Biosystem GmbH). NCM460 cells were incubated with *Lpb. plantarum* strains for 4 h, then they were treated for 24 h with human cytokines mix (IL-1β, TNF-α, and INF-γ) to induce the inflammation^29^. Finally, the supernatants were collected and analysed. Briefly, 50 µl of supernatants were placed on the planar surface of LUNARIS BioChip wells, harboring thousands of microbeads coated with different antibodies, for the simultaneous determination of multiple cytokines. A calibration curve for each cytokines was constructed and used to compute samples concentrations. Inflamed NCM460 cells without bacterial pre-treatment were included in the study as control.

### Data analysis

Results were expressed as mean ± SEM of triplicate experiments. Data were analyzed by means of Prism 7.0 program (GraphPad Software Inc., La Jolla, CA, United States) using the one-way analysis of variance (ANOVA) followed by Bonferroni’s post hoc analysis. A level of *p* < 0.05 was considered statistically significant. Cytokines data were assessed by Student’s *t*-test, with a level of *p* < 0.05 considered statistically significant.
